# Investigation of inter-rater and test-retest reliability of Y balance test in college students with flexible flatfoot

**DOI:** 10.1186/s13102-024-00819-3

**Published:** 2024-02-08

**Authors:** Yalin Zheng, Renzhi Feng, Weiyin Hu, Peng Huang

**Affiliations:** School of Sports Medicine and Rehabilitation, Beijing Sports University, No.48 Xinxi Road, Haidian District, 100084 Beijing, China

**Keywords:** Flexible flatfoot, College Students, Y Balance Test Lower quarter, Reliability

## Abstract

**Background:**

The Lower Quarter Y Balance Test (YBT-LQ) has been widely used to assess dynamic balance in various populations. Dynamic balance in flexible flatfoot populations is one of the risk factors for lower extremity injuries, especially in college populations in which more exercise is advocated. However, no study has demonstrated the reliability of the YBT-LQ in a college student flexible flatfoot population.

**Methods:**

A cross-sectional observational study. 30 college students with flexible flatfoot were recruited from Beijing Sports University. They have been thrice assessed for the maximal reach distance of YBT under the support of the lower limb on the flatfoot side. Test and retest were performed with an interval of 14 days. The outcome measures using the composite score and normalized maximal reach distances in three directions (anterior, posteromedial, and posterolateral). The relative reliability was reported as the Intraclass Correlation Coefficient (ICC). Minimal Detectable Change (MDC), Smallest worthwhile change (SWC), and Standard Error of Measurement (SEM) were used to report the absolute reliability.

**Results:**

For inter-rater reliability, the ICC values for all directions ranged from 0.84 to 0.92, SEM values ranged from 2.01 to 3.10%, SWC values ranged from 3.67 to 5.12%, and MDC95% values ranged from 5.58 to 8.60%. For test-retest reliability, the ICC values for all directions ranged from 0.81 to 0.92, SEM values ranged from 1.80 to 2.97%, SWC values ranged from 3.75 to 5.61%, and MDC95% values ranged from 4.98 to 8.24%.

**Conclusions:**

The YBT-LQ has “good” to “excellent” inter-rater and test-retest reliability. It appears to be a reliable assessment to use with college students with flexible flatfoot.

**Trial registration:**

This trial was prospectively registered at the Chinese Clinical Trial Registry with the ID number ChiCTR2300075906 on 19/09/2023.

## Background

Flexible flatfoot (FFF) is a common musculoskeletal pathology characterized by a collapsed medial longitudinal arch, forefoot abduction and pronation, and hindfoot valgus in the weight-bearing position, which recovers to normal in the non-weight-bearing [1]. Previous studies demonstrated the prevalence of flatfoot between 19.0%∼26.5% in various ages and populations [2]. Moreover, earlier research indicates that flatfoot can impair the mobility of the medial longitudinal arch and increase foot stress, which can result in posterior tibial muscle dysfunction and patellofemoral instability [3], thus affecting the individual’s proprioception, balance, and sports performance [4, 5]. Abnormal structural alterations in the foot have been recognized as a risk factor for lower limb sports injuries, specifically in individuals with flexible flatfoot. The risk of knee injuries, soft tissue injuries, and medial tibial stress syndrome (MTSS) is raised as a result of these changes [6].

Dynamic balance, which refers to an individual’s capacity to maintain a stable center of gravity while exercising, is recognized as one of the risk factors for lower limb sports injuries. Moreover, it has been suggested that it may be a predictor of injury risk in athletes [7, 8]. The YBT-LQ, derived from the Star Excursion Balance Test (SEBT), is an economical and efficient assessment of dynamic balance. It has gained popularity for its application in injury prevention and screening among athletes [9, 10], particularly for lower limb injuries like ankle and knee injuries [11], Additionally, previous studies have shown the potential of this test to detect balance deficits in patients with low back pain [12]. YBT-LQ not only enhances the reproducibility of SEBT but also addresses common errors in SEBT measurements and standardizes the test procedure. The advantage of YBT-LQ over SEBT lies in its standardized protocol, which enables researchers and clinicians to compare results [13]. Furthermore, it has been observed to exhibit high inter-rater reliability (ICC0.73 ∼ 1.00) and test-retest reliability (ICC0.68 ∼ 0.94) in healthy populations [14].

The physical and mental health of college students tends to continuously decline worldwide [15]. Thus, college students should be encouraged to participate in more physical exercise and enhance their physical and mental fitness, to lay a foundation for their healthy growth and lifelong development [16]. The sport demands unilateral balance and dynamic neuromuscular control. Changes in foot structure can alter contact area, joint motion, and muscle activation strategies in the flexible flatfoot population, which can have a negative impact on balance and increase the risk of lower limb sports injuries [17].

The Y Balance Test (YBT) is a low-cost clinical measure of dynamic balance that simulates the demands of exercise requiring unilateral balance, and it has also been used as a clinical outcome measure to measure functional improvement and guide activity progression after injury [18–20]. The YBT is frequently used as an outcome measure of dynamic balance ability in studies related to dynamic balance in patients with flatfoot and provides a better response to detecting deficits in their dynamic balance [4, 21]. However, there is still a gap in the research on the reliability of the Y balance test in the flatfoot population.

However, the current reliability studies of the YBT-LQ have focused primarily on healthy populations and athletes [22, 23]. Although the YBT is currently considered a reliable tool for measuring dynamic balance and predicting injury risk in healthy populations and athletes, there are differences between these populations and college student flatfoot populations.This may limit the generalisability of these findings to the flexible flatfoot college students. The utility of the YBT-LQ to assess dynamic balance in a college student flatfoot population is currently unknown.

Therefore, this cross-sectional observational study aimed to further investigate the reliability of the YBT-LQ in a flexible flatfoot population of college students. We assumed that the relative reliability reported as ICC would be good, corresponding to an ICC > 0.75 indexed by Koo et al. [24]. The absolute reliability reported in terms of SEM and MDC would be on a similar level as found by Shaffer et al. [25]. The value of SWC would be larger than SEM, and YBT can detect the smallest worthwhile changes.

## Methods

### Study design

This cross-sectional observational study was conducted between September 10 and October 20, 2023, at Beijing Sports University, Beijing, China. The study adhered to STROBE guidelines.

### Sample size calculation

A sample size of at least 30 participants was calculated in advance by PASS 2021(NCSS LLC., Kaysville, U.T., USA) to allow for an inter-rater test-retest reliability study that would result in an ICC of at least 0.75, with an expected 0.9 and beta of 0.8. alpha was 0.05, and the dropout rate was 10%.

### Participants

30 Participants were recruited from the Beijing Sports University through announcements and personal referrals. Participants were eligible for inclusion if they were 17 ∼ 25 years college students; had navicular drop test > 10 mm; had no history of lower limb injury within 6 months; had no history of surgery on hip, knee, and ankle joints; and had no serious medical conditions. Participants were excluded if they had vestibular dysfunction or other diseases that affected balance; taking unknown drugs; rheumatoid, neurological, or other causes that affected the hip and knee joints muscles or function.

Written informed consent was obtained from the participants before the start of the study. The Sports Science Experiment Ethics Committee of Beijing Sport University approved the study protocol (2,023,154 H). This trial was registered at the Chinese Clinical Trial Registry (ChiCTR2300075906) on 19/09/2023.

### Testing procedures

The Guidelines for Reporting Reliability and Agreement Studies were used to implement and report this interrater and test-retest reliability study [26]. To examine the inter-rater and test-retest reliability of the YBT, the extent of agreement and reproducibility were calculated between the measurements by two different raters who were fully experienced in the use of YBT. In the first trial, the participant would perform 6 practice sessions to eliminate the learning effect before the formal YBT-LQ and perform the test by the rater within 20 min of the practice session. Subsequently, after a 20-minute break, the test was administered by another rater. A 14-day interval was set between the test and the retest to prevent actual changes in dynamic balance ability and memory effects.

All raters were pre-trained and proficient in the testing process. Hiding test data immediately after completion of the test so that the rater did not have access to each subject’s previous score to minimize bias. All test data would be double-entered into the computer to ensure proper.

### Navicular drop test

The navicular drop test (NDT) was performed to measure the change in height of the medial longitudinal arch of the foot. The participant seated in a chair with the knee flexed to 90° and the second toe aligned with the knee so that the talonavicular joint was in a neutral position. The height between the ground and the navicular tuberosity was measured and noted by the evaluator using a vernier caliper. Then, the participant stood up and measured the height again. The difference between the height of the navicular tubercle in the non-weight-bearing and weight-bearing positions of the foot was calculated. Repeated the test 3 times on both the right and left sides and take the average value. Navicular drop exceeding 10 mm was diagnostic of a flexible flatfoot [27].

### Leg length measure

Participants were instructed to lie in a supine position. They lifted their hips and returned to the starting position. To make sure the pelvis was in alignment, the participant were passively straightened the legs. The participants’ flexible flatfoot side leg length was measured centimeters from the anterior superior iliac spine (ASIS) to the most distal part of the medial malleolus using a cloth tape. This measurement was used to normalize the maximal reach distance. The leg length measurement was carried out before the first trial.

#### Y balance test lower quarter

The test was carried out by the standardized YBT methodology advised by Plisky et al. [13]. The trial protocol was conducted on the side leg with the flexible flatfoot, which was the only difference from the YBT-LQ. The YBT-LQ was completed using the Y Balance Test Kit. Before the test, participants were shown the YBT demonstration video and provided detailed instructions on how to take it. According to the previous research protocol [28–30], the practice phase consisted of six trials in three different directions in order to minimize the learning effect. Participants performed the YBT-LQ at least 3 times and up to 6 times in each direction after the practice phase. All tests were conducted with participants standing barefoot on the pedals, with their single lower limb supported by the flatfoot side. The distal end of the second toe of the supporting leg was located behind the red indicator line, and the contralateral lower limb was extended in three directions, pushing the rectangular indicator block as far as possible with the tip of the foot. Then, recorded the reading of the proximal end of the rectangular block to the nearest 0.5 cm. The outcome of that test was deemed invalid if participants were (1) unable to controllably return to the starting position, (2) accelerate the rectangular block with the outstretched foot to move it farther, (3) touch the ground with their forefoot, (4) or contact the top of the rectangular block for support. The maximal reach distance in each direction was recorded for the 3 valid tests.

The normalized maximal reach distance per reach direction was calculated as follows (Eq. 1) and used as an outcome measure. Additionally, Filipa et al. [31] supplied a formula (Eq. 2) that was used to determine the normalized composite score (CS).

1 $$\begin{array}{l}Normalized{\rm{ }}\ maximal{\rm{ }}\ reach{\rm{ }}\ distance{\rm{ }}\left( {\% {\rm{ }}\ leg{\rm{ }}\ length{\rm{ }}\left[\ {LL} \right]} \right){\rm{ }} = {\rm{ }}\\\left( {absolute{\rm{ }}\ maximal{\rm{ }}\ reach{\rm{ }}\ distance{\rm{ }}\left[ {cm} \right]} \right){\rm{ }}/{\rm{ }}LL{\rm{ }}\left[ {cm} \right]){\rm{ }} \times {\rm{ }}\ 100\% \end{array}$$

2 $$\begin{array}{l}Composite{\rm{ }}\ score{\rm{ }}\left( {\% {\rm{ }}LL} \right){\rm{ }} = {\rm{ }}\\\left\{ {\left( {AT{\rm{ }} + {\rm{ }}PM{\rm{ }} + {\rm{ }}PL} \right){\rm{ }}/{\rm{ }}\left( {leg{\rm{ }}\ length{\rm{ }} \times {\rm{ }}3} \right)} \right\}{\rm{ }} \times {\rm{ }}100\% \end{array}$$

### Statistical analysis

Reliability refers to the consistency of a test or measurement [32]. Calculating the ICC can demonstrate relative reliability, which is the degree to which individuals maintain their location in a sample with repeated measurements [33]. The maximal reach distance in each direction and composite score were calculated as absolute values and values normalized to leg length. The relative reliability of the maximal reach distance in each direction and of the composite score was analyzed by calculating ICC. The inter-rater reliability was determined using the ICC(2,1) and the test-retest reliability was determined using the ICC(3,1).ICC< 0.50 was considered “poor”, 0.50<ICC< 0.75 was considered “moderate”, 0.75<ICC< 0.9 was considered “good”, and ICC ≥ 0.90 was considered “excellent” [24].

The absolute reliability of the data was assessed using the standard error of measurement (SEM) that estimates the amount of error related to the measurement (Eq. 3). Moreover, the minimal detectable change (MDC95%) and the smallest worthwhile change(SWC) were calculated using the formula (Eq. 4 and Eq. 5) to ensure the differences between test and re-test measurements were real and outside the error range [24, 34]. It is worth mentioning that the study was conducted on a population of college students with flexible flatfoot, and there were no strict inclusion requirements for the fitness status of the participants, so the SWC calculated by 0.6 multiplied by SD is relevant for both high and low fitness level participants [34]. For all analyses, the Statistical Package for the Social Sciences (SPSS version 27.0) was used (IBM Corp., Armonk, N.Y., USA).

3 $$SEM = SD * \sqrt {1 - {\rm{ICC}}}$$

4 $$MDC95\% = 1.96 * \sqrt 2 * SEM$$

5 $$SWC = 0.6*SD$$

Bland-Altman plot is a simple and intuitive graphical method of responding to data agreement. The 95% limits of agreement (LOA) was defined as two standard deviations above and below the mean of the difference scores. Plotting the difference between the YBT-LQ and comparison test values versus the mean of the YBT-LQ and comparison test scores yielded Bland-Altman plots with 95% limits of agreement, which were used to visually display measurement errors against true values. Statistical analyses were performed using MedCalc, version 19.4 for Windows (MedCalc Software, Ostend, Belgium).

## Results

30 college students(16 females, 14 males;21.53 ± 0.32years)participated in this study. The characteristics of the participants are shown in Table 1. No adverse events occurred during this study. There was no missing data in the current study.

**Table 1:** Characteristics for participants performing the Y Balance Test

	Mean ± SD[95%CI]	Minimum;Maximum
Sex, n (%)	female 16(53.3%), male14(46.7%)	-
Age(years)	21.53 ± 0.32[20.88,22.18]	18;24
Height(cm)	170.47 ± 8.75[167.19,173.73]	156;186
Mass(kg)	64.65 ± 12.21[60.09,69.21]	44;87
BMI (kg/m²)	22.09 ± 2.75[21.06,23.11]	15.59;25.31
Length(cm)	85.04 ± 5.02[83.16,86.91]	75.3;92.2
Flexible flatfoot, n (%)	Left 6(20%), Right24(80%)	-

Values are presented with mean ± standard deviation (SD) and 95% confidence intervals [95% CI].BMI = body mass index

The absolute and normalized maximal reach distance at the test for two raters and retest in the three test directions (anterior, posteromedial, and posterolateral) and the composite score are present in Table 2.

**Table 2:** The absolute and normalized maximal reach distance at test and retest in the three test directions (anterior, posteromedial, and posterolateral) and the composite score

Direction	Absolute Maximal Reach Distance(cm)Mean ± SD [95%CI]	Normalized Maximal Reach Distance (%) Mean ± SD[95%CI]
Rater A		
AT	58.65 ± 5.16[56.72,60.58]	69.11 ± 6.25[66.77,71.44]
PM	97.6 ± 9.99[93.87,101.33]	114.74 ± 9.15[111.33,118.16]
PL	94.32 ± 8.83[91.02,97.61]	110.97 ± 8.83[107.67,114.26]
CS	250.57 ± 20.16[243.04,258.09]	98.27 ± 6.37[95.89,100.64]
Rater B		
AT	57.80 ± 5.55[55.73,59.87]	68.14 ± 7.10[65.49,70.49]
PM	96.8 ± 8.70[93.55,100.05]	113.85 ± 7.95[110.88,116.82]
PL	94.08 ± 8.20[91.02,97.14]	110.73 ± 8.38[107.60,113.86]
CS	248.68 ± 17.94[241.99,255.38]	97.57 ± 5.95[95.35,99.79]
Retest		
AT	58.15 ± 5.17[56.22,60.08]	68.54 ± 6.43[66.14,70.94]
PM	97.07 ± 9.89[93.37,100.76]	114.16 ± 9.71[110.54,117.79]
PL	93.32 ± 8.65[90.09,96.55]	109.83 ± 9.03[106.45,113.20]
CS	248.53 ± 18.62[241.58,255.49]	97.51 ± 6.20[95.19,99.82]

Values are presented with mean ± standard deviation (SD) and 95% confidence intervals [95% CI]

Normalized reach distance was calculated as (absolute reach distance/leg length) *100%

AT = anterior; PM = posteromedial; PL = posterolateral CS = composite score

For inter-rater reliability, the ICC values for all directions ranged from 0.84 to 0.92, SEM values ranged from 2.01 to 3.10%, SWC values ranged from 3.67 to 5.12%, and MDC95% values ranged from 5.58 to 8.60% (Table 3). For test-retest reliability, the ICC values for all directions ranged from 0.81 to 0.92, SEM values ranged from 1.80 to 2.97%, SWC values ranged from 3.75 to 5.61%, and MDC95% values ranged from 4.98–8.24%(Table 4).

**Table 3:** Inter-rater reliability for the YBT-LQ in the flexible flatfoot college students

YBT-LQ	ICC (2,1)	95%CI	SEM (%)	SWC (%)	MDC95% (%)
AT	0.84	[0.69,0.92]	2.67	3.99	7.39
PM	0.92	[0.84,0.96]	2.44	5.11	6.75
PL	0.87	[0.74,0.94]	3.10	5.12	8.60
CS	0.89	[0.79,0.92]	2.01	3.67	5.58

ICC: Intra Class Correlation; 95% CI: 95% confidence interval; SEM: Standard Error of Measurement; SWC: Smallest worthwhile change; MDC95%: Minimal detectable change using a 95% confidence interval

**Table 4:** Test-retest reliability for the YBT-LQ in the flexible flatfoot college students

YBT-LQ	ICC (3,1)	95%CI	SEM (%)	SWC (%)	MDC95% (%)
AT	0.81	[0.63,0.90]	2.76	3.78	7.64
PM	0.90	[0.80,0.95]	2.97	5.61	8.24
PL	0.89	[0.79,0.95]	2.92	5.32	8.08
CS	0.92	[0.83,0.96]	1.80	3.75	4.98

ICC: Intra Class Correlation; 95% CI: 95% confidence interval; SEM: Standard Error of Measurement; SWC: Smallest worthwhile change; MDC95%: Minimal detectable change using a 95% confidence interval

**Fig. 1:**
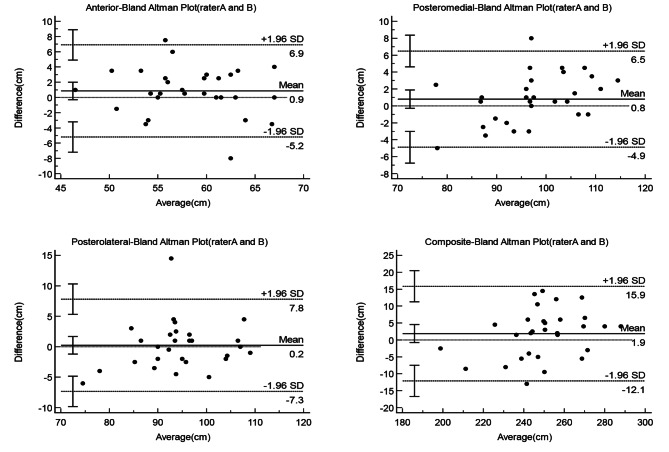
Bland-Altman Plots with 95% limits of agreement illustrate measurement errors against true values by plotting each of the three YBT-LQ directions (anterior, posteromedial, and posterolateral) and the composite score differences between the two raters. Plot of the mean (x-axis) of the measurements of two raters against the difference (y-axis) between two raters

**Fig. 2:**
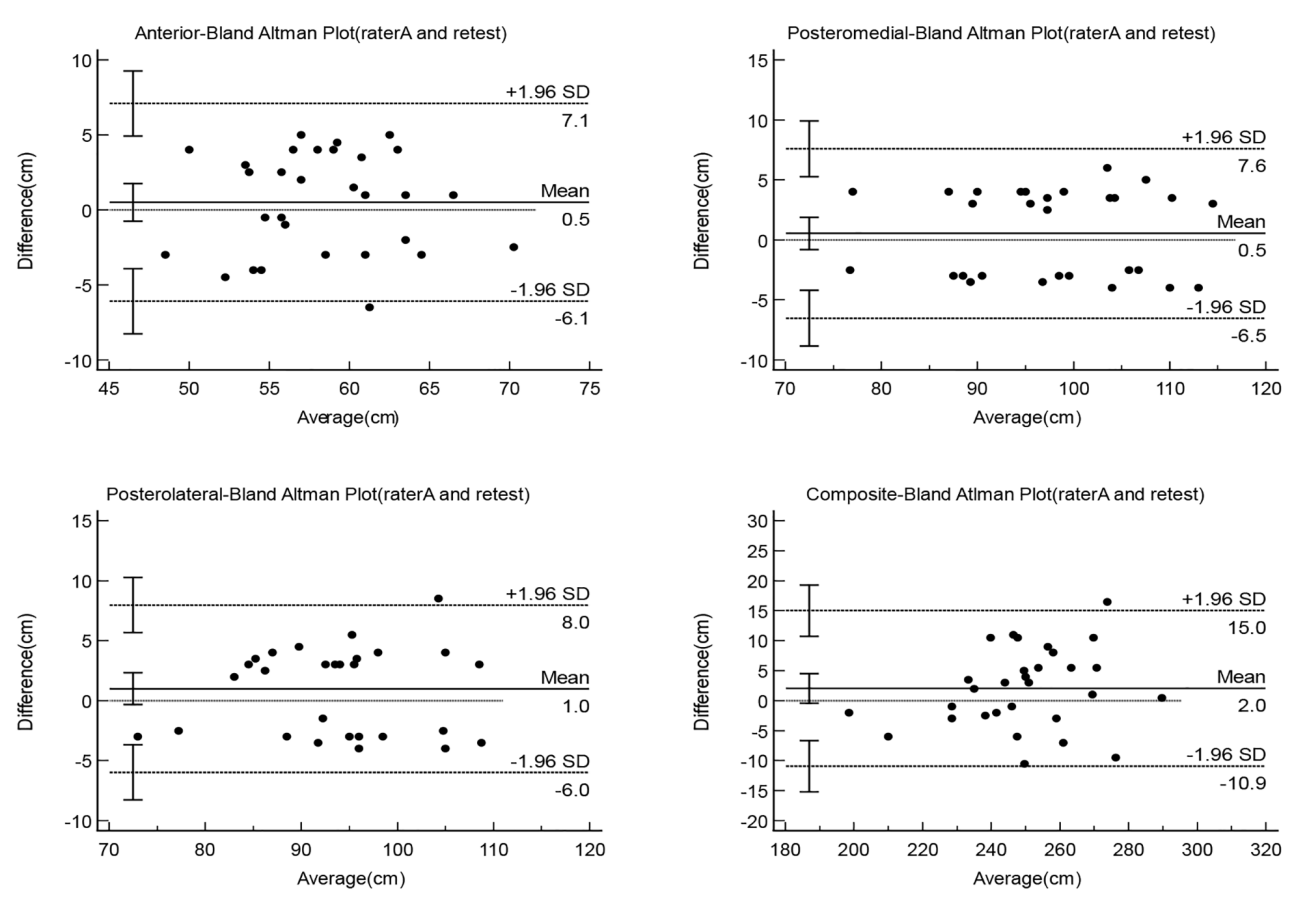
Bland-Altman Plots with 95% limits of agreement illustrating measurement errors against true values by plotting each of the three YBT-LQ directions (anterior, posteromedial, and posterolateral) and the composite score differences between the rater A test and retest. Plot of the mean (x-axis) of the measurements of test and retest against the difference (y-axis) between test and retest

The two lines above and below the mean difference in the Bland Altman plot represent the 95% limits of agreement(95%LOA), indicating the size of the measuring errors. The Bland Altman plots showed that almost all points are within the 95%LOA. For each of the three test directions and the composite score, the difference between the two raters and test-retest measurements plotted against the mean difference was close to zero (Figs. 1 and 2).

## Discussion

To the best of our knowledge, this is the first study to examine the reliability of the YBT-LQ in a population of college students with flexible flatfoot. This study provides preliminary evidence of the reliability of the YBT for use with a population of college students with flexible flatfoot, and the results of the study indicate that the YBT-LQ has “good” to “excellent” inter-rater and test-retest reliability. The inter-rater point estimates for ICCs ranged from “moderate” to “excellent” on the anterior (Table 3, first line, [0.69–0.92]) and posterolateral (Table 3, third line, [0.74–0.94]), ranged from “good” to “excellent” on the posteromedial (Table 3, second line, [0.84–0.96]) and the composite scores (Table 3, fourth line, [0.79–0.92]).The test-retest point estimates for ICCs ranged from “moderate” to “excellent” on the anterior (Table 4, first line, [0.63–0.90]), ranged from “good” to “excellent” on the posteromedial (Table 4, second line, [0.80–0.95]), posterolateral (Table 4, third line, [0.79–0.95]) and the composite scores (Table 3, fourth line, [0.83– 0.96])..Moderate to excellent inter-rater, test-retest reliability has been reported in previous research among 51 health populations aged 19 ∼ 50 years old (ICC0.79 ∼ 0.86, SEM2%∼4%) [28]; 178 teenagers aged 11 ∼ 19 years old(ICC0.4 ∼ 0.96, SEM1.77%∼5.81%) [35]; 110 high school athlete(ICC0.63 ∼ 0.89, SEM1.94%∼4.17%) [36]. These results support the findings of our study and import the YBT-LQ as a valid test method for detecting changes in dynamic equilibrium over time at the group level.

Absolute reliability is the degree of variability of an individual’s repeated measurements. The SEM, SWC, and MDC 95% were calculated to quantify the error due to repeated measurements. Lower SEM and MDC 95% suggest strong reliability of the measurements [33]. It can be said that the measurement error is minimal and the measurement is reliable when the SEM value is less than 10% of the highest or average measurement value [37]. The inter-rater and test-retest have a rather smaller SEM of the YBT-LQ between 1.80 and 3.10%. SWC ranged from 4.98 to 8.60%, all greater than SEM, indicating that the Y balance test can detect smallest worthwhile changes. Further MDC95%, which represents the change needed to identify clinically relevant effects between repeated measures ranged from 4.98 to 8.60%. Compared to other studies, our MDC95% values are close to those(5%∼11%)reported by Foldager et al. [28]. The difference between our values and those of Schwiertz et al. [36] might be due to the investigated cohort. Our study was in a college student’s cohort, while Schwiertz investigated 178 adolescents in grades 6–11, who may have shown the worse ability to understand and perform YBT compared to college students. Notably, the composite score showed the most reliable results with the higher ICC and the lowest SEM and MDC95% for both inter-rater and test-retest. Therefore, it is recommended that the composite score of the YBT be used in screening to quickly assess dynamic balance in a college student population with flexible flatfoot.

However, this study has some limitations. Because raters were involved in the research design process, the Hawthorne effect (i.e., raters were unaware that their judgments would be compared with those of other assessors) could not be avoided and the raters’ behavior may be altered as a result of the awareness of being observed. Moreover, for the test and retest reliability test, we were unable to blind the rater to the fact that he was going to take the YBT-LQ test. However, we believe the potential for a recall bias was held to a minimum due to several factors: (1) Hiding test data immediately after completion of the test so that the rater does not have access to each subject’s previous score; (2) the data were not analyzed until the end of the study; and (3) the testing and retesting intervals 14 days, with as many as 12 participants being tested on a single day. Given these factors, it is unlikely that the rater had a clear recall of any individual’s last test performance. Additionally, the findings of the study revealed that the posterior lateral side had a higher inter-rater measurement error (SEM3.10%) than did the other two directions (SEM2.44 ∼ 2.67%), which may be since only the lower limb on the side of the flatfoot was tested in this study, whereas the YBT protocol recommends switching between the right and left leg in each test direction. Prolonged single-leg support may increase the possibility of muscle fatigue, which would decrease the dynamic balance [38]. The intervals between each testing direction could be appropriately lengthened for subjects in subsequent investigations to reduce this measurement error. Finally, although previous research protocols have shown that six practice sessions in each direction before the start of the formal test can minimize the learning effect, a paired-samples t-test would have made this experimental protocol more rigorous. We hope that future researchers will improve on the experimental protocol of this study by fully recording the values of the three valid experiments and conducting a paired-samples t-test (trial-by-trial values) to explore whether six practice sessions to minimize the learning effect is reliable and provide more reliable evidence for the application of the YBT-LQ.

In summary, this study demonstrates that the YBT-LQ has “good” to “excellent” intra-rater and inter-rater reliability as a simple, rapid method of assessing dynamic balance ability in a college student flatfoot population. It can be applied to screen this population in college so that targeted rehabilitation training and education in advance can be provided to avoid sports injuries and improve the physical fitness of college students. However, distance-attainment criteria have not yet been developed in various populations. Future research could focus on the reliability of the YBT and the validity of the YBT in predicting sports injuries using standardized test protocols in different clinical settings and populations. In addition, different distance-to-reach standards could be developed for different groups through standardized data collection and prospective studies.

## Conclusions

The inter-rater and test-retest reliability of YBT-LQ ranges from “good” to “excellent” as a reliable tool for assessing the dynamic balance ability of college students with flexible flatfoot populations.

## Data Availability

The data generated and analyzed during the present study are not publicly available due to ethical restrictions but are available from the corresponding author upon reasonable request.

## Abbreviations

YBT-LQ: Lower Quarter Y Balance Test
AT: Anterior
PM: Posteromedial
PL: Posterolateral
CS: Composite score
BMI: Body mass index
NDT: The navicular drop test
ICC: Intra Class Correlation
SEM: Standard Error of Measurement
SWC: Smallest worthwhile change
MDC95%: Minimal detectable change using a 95% confidence interval
